# Molecular dynamics simulations highlight structural and functional alterations in deafness–related M34T mutation of connexin 26

**DOI:** 10.3389/fphys.2014.00085

**Published:** 2014-03-04

**Authors:** Francesco Zonta, Damiano Buratto, Chiara Cassini, Mario Bortolozzi, Fabio Mammano

**Affiliations:** ^1^Dipartimento di Fisica e Astronomia “G. Galilei”, Università degli Studi di PadovaPadova, Italy; ^2^Istituto Veneto di Medicina Molecolare, Fondazione per la Ricerca Biomedica AvanzataPadova, Italy; ^3^Istituto di Neuroscienze, Consiglio Nazionale delle RicerchePadova, Italy

**Keywords:** genetic deafness, conductance, gating, gap junction channels, potential of mean force, mean first passage time

## Abstract

Mutations of the GJB2 gene encoding the connexin 26 (Cx26) gap junction protein, which is widely expressed in the inner ear, are the primary cause of hereditary non-syndromic hearing loss in several populations. The deafness–associated single amino acid substitution of methionine 34 (M34) in the first transmembrane helix (TM1) with a threonine (T) ensues in the production of mutant Cx26M34T channels that are correctly synthesized and assembled in the plasma membrane. However, mutant channels overexpressed in HeLa cells retain only 11% of the wild type unitary conductance. Here we extend and rationalize those findings by comparing wild type Cx26 (Cx26WT) and Cx26M34T mutant channels *in silico*, using molecular dynamics simulations. Our results indicate that the quaternary structure of the Cx26M34T hemichannel is altered at the level of the pore funnel due to the disruption of the hydrophobic interaction between M34 and tryptophan 3 (W3) in the N–terminal helix (NTH). Our simulations also show that external force stimuli applied to the NTHs can detach them from the inner wall of the pore more readily in the mutant than in the wild type hemichannel. These structural alterations significantly increase the free energy barrier encountered by permeating ions, correspondingly decreasing the unitary conductance of the Cx26M34T hemichannel. Our results accord with the proposal that the mutant resides most of the time in a low conductance state. However, the small displacement of the NTHs in our Cx26M34T hemichannel model is not compatible with the formation of a pore plug as in the related Cx26M34A mutant.

## Introduction

Connexins are integral transmembrane proteins that form intercellular channels in vertebrates. Six connexins form a hexamerical assembly, known as connexon or hemichannel, which delineates an aqueous pore with a minimum diameter of ~1.2 nm. When two hemichannels from adjacent cells dock and join, leaving a gap of ~2–3 nm, they may form an intercellular gap junction channel which spans the two plasma membranes and allows the exchange of cytoplasmic molecules with size up to ~1 kDa (Goodenough and Paul, 2009). The importance of electrical and molecular signaling through gap junction channels is widely recognized (Evans et al., 2006; Harris, 2007). It is also well established that connexin hemichannels open in response to various types of stimuli and conditions, including mechanical, shear, ionic and ischemic stress and provide a pathway for the release of intracellular ATP, glutamate, NAD^+^ and prostaglandin E2, which act as paracrine messengers (Evans et al., 2006).

Virtually all cells in solid tissues are coupled by gap junctions (Goodenough and Paul, 2009), thus it is not surprising that mutations in connexin genes have been linked to a variety of human diseases, including cardiovascular anomalies, peripheral neuropathy, skin disorders, cataracts, and deafness (Wei et al., 2004; Laird, 2006; Dobrowolski and Willecke, 2009). Of notice, about half of all cases of human deafness in countries surrounding the Mediterranean have been linked to mutations in the *GJB2* gene, which encodes Cx26 (Zelante et al., 1997; Petit et al., 2001). In this paper, we focus on hemichannels formed by the deafness–associated Cx26M34T mutant. According to the published X–ray model of the human Cx26WT gap junction channel (Maeda et al., 2009), M34 interacts with W3 of the NTH belonging to an adjacent connexin. The six NTHs fold inside the pore and the M34–W3 hydrophobic interactions stabilize their position at the cytoplasmic mouth (see Figure 5 of Maeda et al., 2009).

The Cx26M34T mutant, which encodes full–length products, was originally described by Kelsell et al. (1997) who associated it with a dominant form of non-syndromic deafness (DFNA3) and also noted that M34 is conserved across several species both in Cx26 and in the closely related connexin 32 (Cx32) protein. Cx26M34T was also linked to a recessive form of hearing loss by Houseman et al. (2001). However, subsequent studies on the family first described by Kelsell et al. (1997) uncovered the association of dermatological signs in deaf patients and identified another dominant mutation in *GJB2* segregating with the disease, casting doubts on the significance of the Cx26M34T variant. Other authors reported normal hearing in heterozygous carriers of Cx26M34T associated with either Cx26G35del or other Cx26 recessive mutations (Denoyelle et al., 1997; Kelley et al., 1998; Scott et al., 1998; Feldmann et al., 2004) and classified it as a benign polymorphism. An attempt to rationalize these results noted that, even though Cx26M34T is significantly overrepresented among patients, its relative penetrance is about 1/10 of that of undisputedly pathogenic mutations (Pollak et al., 2007).

The functional expression of the Cx26M34T connexin in *Xenopus* oocytes showed that the mutant exerts a dominant negative effect on Cx26WT (White et al., 1998). Later on Thonnissen et al. (2002) observed low levels of dye transfer between HeLa cells overexpressing Cx26M34T, providing the first evidence that this mutant could traffic to the cell membrane and form intercellular channels in a mammalian expression system, albeit with reduced efficiency. In contrast, Oshima et al. (2003) reported that assembly of Cx26M34T in HeLa and Sf9 cells resembles that of Cx26WT and that dye transfer in these cells is close to normal. Other electrophysiological studies performed in paired *Xenopus* oocytes concluded that Cx26M34T was capable of forming functional heterotypic channels with Cx32, albeit with abnormal gating properties (Skerrett et al., 2004). Based on these results, it was suggested that Cx26M34T/Cx32 heterotypic channels are not fully open at rest but are activated when positive transjunctional voltages are applied to the Cx26M34T side (Skerrett et al., 2004).

The pathogenetic role of M34T was confirmed in a study by Bicego et al. (2006) showing that, at a cellular level, Cx26M34T is correctly synthesized and targeted to the plasma membrane in HeLa cells, but inefficiently forms intercellular channels that display an abnormal electrical behavior and retain only 11% of the unitary conductance of Cx26WT. Moreover, Cx26M34T channels failed to support the intercellular diffusion of fluorescent tracers and the spreading of mechanically induced intercellular Ca^2+^ waves.

In the strictly homologous Cx32 protein, several mutations of Met34 have been associated with X linked Charcot–Marie–Tooth disease (M34T, M34I Tan et al., 1996, M34V Latour et al., 1997, M34K Yum et al., 2002). Single–channel recordings performed in transfected N2A cells showed that Cx32M34T mutants reside in a low–conductance (15 pS) substate 98% of the time at −80 mV (Oh et al., 1997).

Here, starting from previously described molecular models (Zonta et al., 2012, 2013) based on the 3.5 Å X–ray data (Maeda et al., 2009), we constructed a model of the Cx26M34T hemichannel and analyzed it by use of molecular dynamics simulations. The results we present provide an interpretative framework, at atomic scale, of the reduced opening probability and conductance observed in the residual open state of the mutant hemichannel. This work advances our understanding of the molecular mechanisms that underline ion permeation and gating of connexin hemichannels and provides a mechanistic link between connexin mutations and hereditary deafness.

## Methods

### Equilibrium molecular dynamics of Cx26WT and Cx26M34T connexons

We generated the Cx26M34T hemichannel model starting from an equilibrium configuration of the Cx26WT model published in Refs. (Zonta et al., 2012, 2013) and mutating the 34th amino acid of each connexin protomers with the *mutate* tool of the Swiss PDB–Viewer (Guex and Peitsch, 1997). As done previously for Cx26WT, we embedded the Cx26M34T hemichannel in a plasma membrane represented by 494 Palmytoyl Oleoyl Posphatidly Choline molecules (POPC), following the same methodology described in Pantano et al. (2008). In order to achieve a faster convergence of molecular dynamics trajectories, coordinates for the original phospholipid bilayer were obtained from an equilibrium configuration of the membrane model described in Pantano and Carafoli (2007). The system then was solvated with full atom TIP3P water containing Cl^−^ and K^+^ ions at a concentration of ~0.15 M to neutralize the positive net charge of the connexon and to mimic a physiological ionic strength. The whole system comprised 204664 and 205825 atoms respectively for Cx26M34T and Cx26WT simulations.

We initially performed a short energy minimization run, followed by equilibrium molecular dynamics under periodic boundary condition using unitary cells of 12 × 12 × 11 nm for both systems, consistent with the channel density measured in a Cx26 gap–junction plaque by atomic force microscopy (Muller et al., 2002). Equilibrium molecular dynamics simulations, performed with GROMACS 4.6 software (Hess et al., 2008) using the Amber03 force field (Duan et al., 2003) under constant *nPT* conditions, lasted 40 ns, the last 18 ns of which were retained for data analysis. Temperature *T* and pressure *P* were kept constant, at 300 K and 1 atm respectively, using the Berendsen thermostat and barostat (Berendsen et al., 1984). Fast smooth Particle–Mesh Ewald summation (Darden et al., 1993) was used for long–range electrostatic interactions, with a cut off of 1.0 nm for the direct interactions.

Root mean squared deviation (RMSD) of the transmembrane domain stabilized after 15 ns, whereas short range interaction between membrane and protein (short range Lennard Jones plus short range Coulomb potential) stabilized after 20 ns. Moreover, root mean squared fluctuations (RMSF) were well equilibrated in the last 20 ns time window of the simulation (data not shown).

### Estimate of the potential of mean force (PMF) from steered molecular dynamics

**Figure 1:**
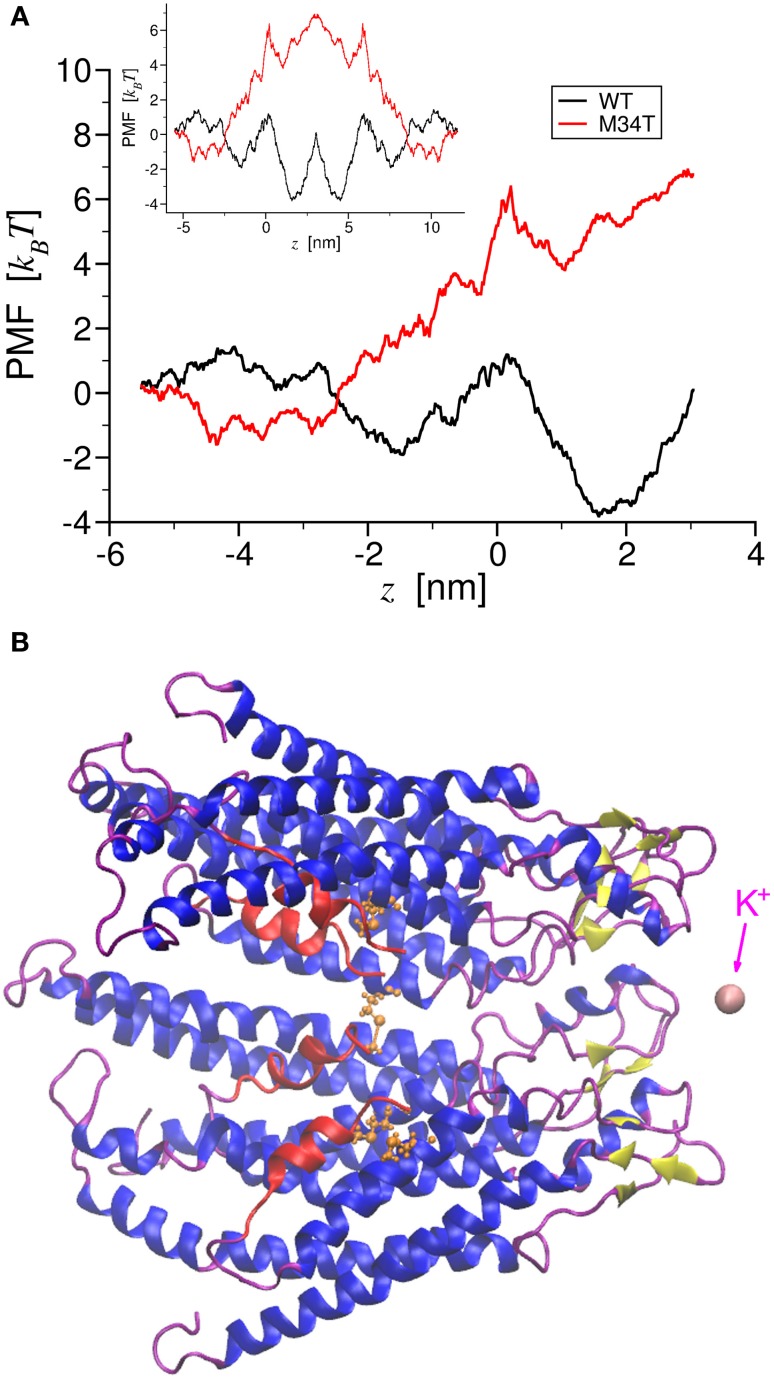
**Potential of Mean Force of potassium ion permeation through Cx26WT and Cx26M34T**. Panel **(A)** shows the total PMF for a single K^+^ ion permeating through the two different hemichannels as a function of the pore axial coordinate. The black trace corresponds to the wild type, while the red trace to the mutant. The inset shows the corresponding PMFs for the entire gap junction channel. In panel **(B)**, a cartoon representation of a section made of four connexins protomers of the Cx26M34T hemichannel is drawn in register with the axis coordinate in A. The NTHs are colored in red, while in orange we show a ball and stick representation of Thr34. This also frame shows the K^+^ ion (in pink) at the final position, just outside the extracellular mouth of the hemichannel. Note that the two PMFs diverge largely in the region around the NTHs.

Simulations were performed under constant volume conditions on the previously equilibrated systems. To force the passage of a K^+^ ion through the channel pore (Figure 1), we connected it to one end of a linear spring with elastic constant *k* of 2000 kJ mol^−1^nm^−2^ and zero resting length. The other end of the spring shifted along pore axis (*z* direction) from the cytoplasmic to the extracellular side of the hemichannel at a constant velocity of 0.5 nm ns^−1^. The shifting spring was stiff enough to keep the K^+^ in the proximity of the *z* axis, with a standard deviation of 0.034 nm. The simulations spanned a total of 8.6 nm in 17.2 ns for each system. The mean force *F*(*x, y, z*) exerted on the ion by the hemichannel amino acids was gauged by the instantaneous spring extension averaged over 40 ps time intervals.

*F*(*x, y, z*) balanced effectively all other forces acting on the ion at each point along the K^+^ trajectory, therefore the work profile

(1) $$W_{HC}(z)=\int_{0}^{z}F_{z}\;(\text{0},\text{0},\zeta )\;d\zeta$$

which is known as the PMF, has the meaning of a free energy profile for the permeation of a single K^+^ ion (Kirkwood, 1935; Roux and Karplus, 1991a,b; Park and Schulten, 2004) through the hemichannel (HC) pore (Figure 1A). We derived the PMF for the full gap junction channel *W_GJ_*(*z*) (Figure 1A, inset) by reflecting *W_HC_*(*z*) about a vertical axis passing through the *z* coordinate corresponding to extracellular end of the hemichannel (Zonta et al., 2013).

### Computation of ionic conductance

To link quantitatively PMF and ionic conductance, let us consider the first time an unforced K^+^ ion reaches one end the full gap junction channel (labeled *r*) given that it started at point (0, 0, *z*_0_). Such *first passage time* of the permeation process has mean value τ given by:

(2) $$\tau =\frac{1}{D}\int_{z_{0}}^{r}e^{\beta W_{GJ}(y)}dy\int_{l}^{y}e^{-\beta W_{GJ}(z)}dz$$

which can be computed provided the PMF *W_GJ_*(*z*) and the bulk diffusion coefficient *D* are known (Szabo et al., 1980; Zwanzig, 1988). In Equation 2 the factor β = (*k_B_ T*)^−1^, where *T* is absolute temperature and *k_B_* is the Boltzmann constant. The numerical value we used for *D* = 1.957 · 10^−9^ *m*^2^/ s was experimentally determined in Samson et al. (2003) and accords with an independent estimate we obtained from molecular dynamics simulations of K^+^ in the bulk.

If a single ion were to occupy the pore at any time, the transition rate would be

(3) $$\kappa =\frac{1}{\tau }$$

and net unitary current could be estimated as the difference between forward (*κ _l → r_*) and reverse (*κ _r → l_*) transition rates multiplied by the charge *q* of the ion:

(4) $$I=q(\kappa_{l\to r}-\kappa_{r\to l})$$

In equilibrium conditions the current is obviously null. Therefore, to compute the unitary conductance

(5) $$\gamma_{0}=\frac{I}{\Delta V}$$

an electrical potential difference Δ*V* must be applied between the two ends of the channel. Assuming that the electrical potential is a linear function of the pore axial coordinate *z* (Tao et al., 2012), we can take its presence into account by adding a suitable term to the PMF, yielding a new function

(6) $$U(z)=W_{GJ}(z)+\frac{q\Delta V}{L}(L-x)$$

After replacing *W_GJ_*(*z*) with *U*(*z*) in Equation 2, we numerically computed the forward and reverse transition rates as:

(7a) $$\kappa_{l\to r}={\left[\frac{1}{D}\int_{z_{0}=l}^{r}e^{\beta U(y)}dy\int_{l}^{y}e^{-\beta U(z)}dz\right]}^{-1}$$

(7b) $$\kappa_{r\to l}={\left[\frac{1}{D}\int_{z_{0}=r}^{l}e^{\beta U(y)}dy\int_{r}^{y}e^{-\beta U(z)}dz\right]}^{-1}$$

for ten different values of the imposed Δ*V*. For each value, we estimated the net current *I* using Equation 4. We finally plotted *I* vs. Δ*V* and derived γ_0_ as the slope of the interpolating line.

### Statistical analysis

Means are quoted ± standard error of the mean (s.e.m.) and *p-values* are indicated by letter *p*. Statistical comparisons were made using the Mann–Whitney *U*-test (Mann and Whitney, 1947); *p* < 0.05 was selected as the criterion for statistical significance.

## Results

### Molecular dynamics predicts conductance values that accord with experimental results for both Cx26WT and Cx26M34T

To gain insight into the role played by the M34T mutation, we used steered molecular dynamics to derive the PMFs for K^+^ ion permeation (Figure 1; see Methods). The PMF profiles for Cx26WT and Cx26M34T differ primarily in the narrowing region of the pore (Figure 1A), where the six NTHs fold inside the cytoplasmic mouth of the hemichannel (Figures 1B, Supplementary Movie S1). We used these PMFs to compute the full channel unitary conductance γ_0_ as detailed in the Methods and obtained the values reported in Table 1, first column. Although those figures are one order of magnitude smaller than their respective measured counterparts (Table 1, third column), their ratio *R_MD_* = γ_0, *M*34*T*_/γ_0, *WT*_ = 9.5% is in good agreement with the experimental result (*R_EXP_* = 11.4%) (Bicego et al., 2006).

**Table 1:** **Comparison between conductance predicted by molecular dynamics simulations and experimental values**.

	**Single ion [pS]**	**Multi–ion correction [pS]**	**Experimental value [pS]**
WT	9.64	105	114
M34T	0.92	10	13
Ratio M34T/WT	9.6%	9.6%	11.4%

As detailed in the Methods section, the conductance values we computed were derived from transition rates of ion permeation through the channel assuming that a single ion can occupy the channel at a given time. This assumption is probably unrealistic. Therefore, to obtain a better estimate for the ionic conductance, we contemplated the possibility that *N*_*I*_ K^+^ ions occupy the channel simultaneously. The maximum conductance is achieved when the ions permeate the channel with minimal reciprocal interaction. Indeed, ionic conductance saturates with increasing salt concentration, and, when many ions are allowed to occupy the channel simultaneously, deviation from independent ion transition is observed (Hille and Schwarz, 1978).

To estimate *N*_*I*_, we assumed that electrostatic repulsion is negligible if the ions are found at the relative distance 2λ_*D*_, where

(8) $$\lambda_{D}=\sqrt{\frac{\varepsilon_{0}\varepsilon_{r}k_{B}T}{\text{2}e^{2}N_{A}c}}\approx \text{0}.\text{79 nm}$$

is the Debye length (a Debye sphere is a volume whose radius is the Debye length, outside of which charges are electrically screened; in the formula above, *e* is the proton's charge, *N_A_* is Avogadro's number and *c* the concentration). We then computed

(9) $$N_{I}=\frac{L}{\text{2}\lambda_{D}}=\frac{\text{17}.\text{2 nm}}{\text{1}.\text{58 nm}}\approx \text{10}.\text{89}$$

where *L* is the length of the full gap junction channel. We then re-estimated the unitary conductance as γ = *N_I_*γ_0_ for both Cx26WT and Cx26M34T. The corrected results (Table 1, second column) agree far better with the experimental data (Table 1, third column).

**Figure 2:**
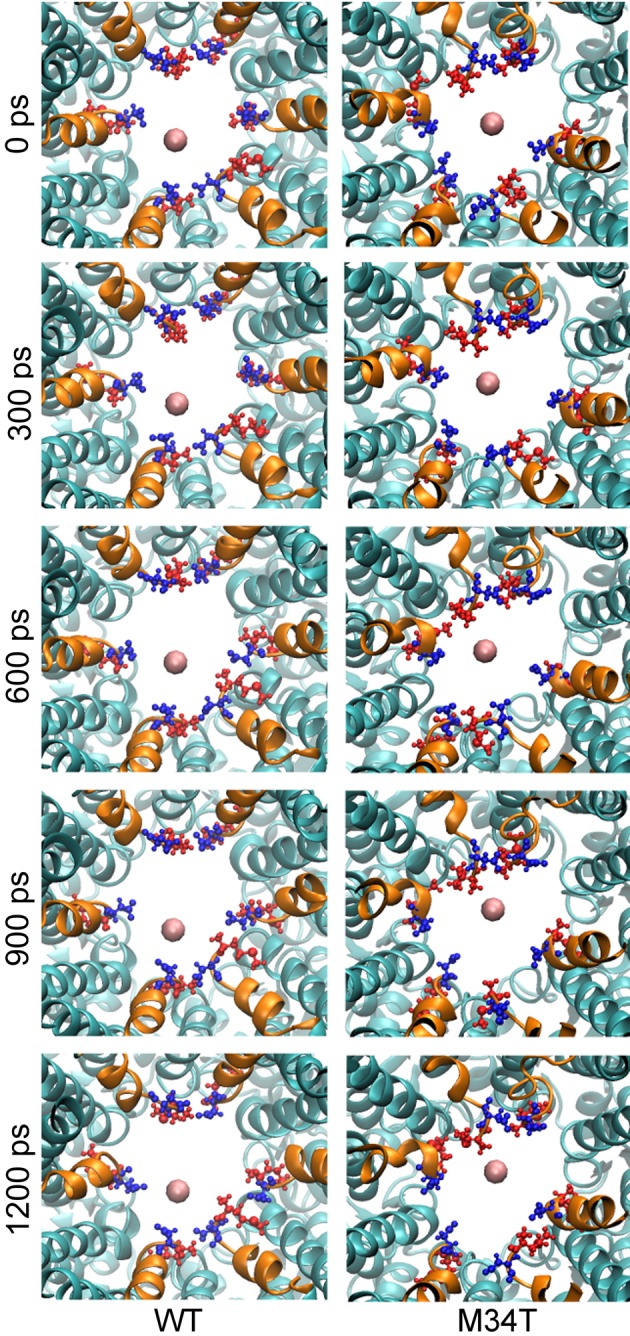
**Snapshots of potassium steered molecular dynamics**. The pictures show, from a cytoplasmic point of view, five different snapshots in a region proximal to the NTHs. The K^+^ ion is shown as a pink sphere and the NTHs are highlighted in orange. M1 (blue) and D2 (red) residues are drawn in ball and stick representation. In the mutant, these residues protrude more toward the center of the pore and consequently the energy of interaction with K^+^ is higher and results in an increased total PMF.

The reduced conductance of Cx26M34T correlates with the observation that the first residues (M1, D2) of the NTHs in the Cx26M34T hemichannel protrude more toward the center of the pore than their WT counterparts and thus interact more efficiently with the drifting K^+^ ions (Figure 2, Supplementary Movie S2).

### Analysis of equilibrium molecular dynamics trajectories reveals an asymmetric configuration of NTHs in Cx26M34T hemichannels

**Figure 3:**
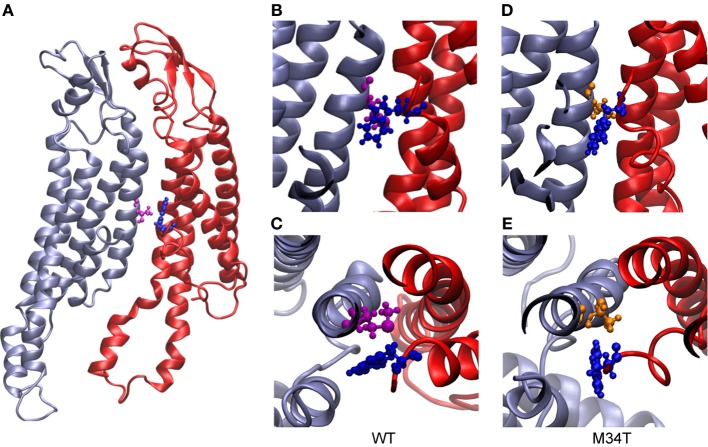
**Structural comparison between Cx26WT and Cx26M34T**. In panel **(A)**, two adjacent wild type connexins are shown in ribbon representation. The two residues highlighted in ball and stick representation are M34 (purple) and W3 (blue). Panels **(B)** (top view) and **(C)** (side view) show details of the hydrophobic interaction between these two residues. **(D)** and **(E)**, same as **(B)** and **(C)** for Cx26M34T; W3 is again represented in blue, while T34 in orange. Note that the interaction present in the wild type is disrupted in the mutant.

**Figure 4:**
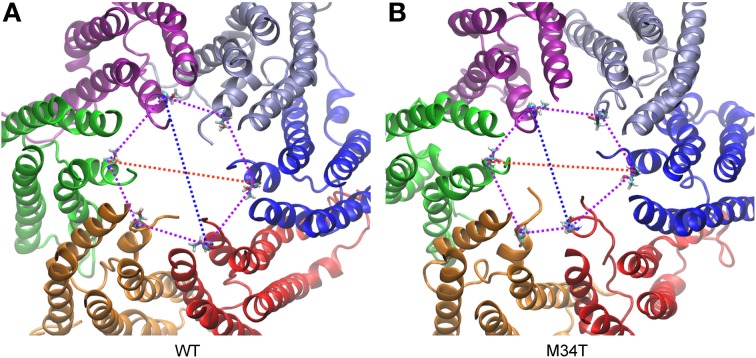
**Analysis of symmetry index. (A)** Cx26WT connexon model. **(B)** Cx26M34T connexon model. Shown are the major (red) and minor (blue) diameter and the angles (purple) of the hexagon built on T5 alpha carbons for a snapshot of the equilibrium dynamics. The six connexins are rendered with different colors and represented in ribbons. Each T5 alpha carbon is represented with its Van der Waals radius, while the rest of the amino acid is represented in licorice.

As shown in Figure 3, replacing a hydrophobic M with a polar T in position 34 disrupts the hydrophobic interaction between M34 and W3 in the NTH of the adjacent connexin. Consequently, the six NTHs of the Cx26M34T hemichannel rearranged in a more asymmetric configuration in the course of the 40 ns equilibration process. To measure these changes and to compare Cx26M34T to Cx26WT quantitatively, let us introduce an *eccentricity coefficient E* that gauges departure from a perfect hexagonal symmetry. Specifically, we define *E* as the ratio between the maximum (*D*) and the minimum (*d*) diameter of a hexagon built on the alpha carbons of corresponding amino acids in the six protomers (Figure 4). We computed *E* for amino acids number 2 to 14 of the NTHs, averaged the results over 100 configurations spanning the last 18 ns of equilibrium molecular dynamics, yielding *E_WT_* = 1.10 ± 0.05 (mean ± s.e.m.) for the Cx26WT hemichannel and *E*_*M*34*T*_ = 1.16 ± 0.11 (mean ± s.e.m.) for the Cx26M34T hemichannel. Since the distributions of *E*-values are not normal, we analyzed the significance level of the observed difference using the Mann Whitney *U*-test (Mann and Whitney, 1947). The *p*-value of 0.03 returned by the test indicates that, compared to Cx26WT, the distribution of Cx26M34T data is significantly shifted toward larger *E* values (i.e., it has a higher degree of asymmetry).

**Figure 5:**
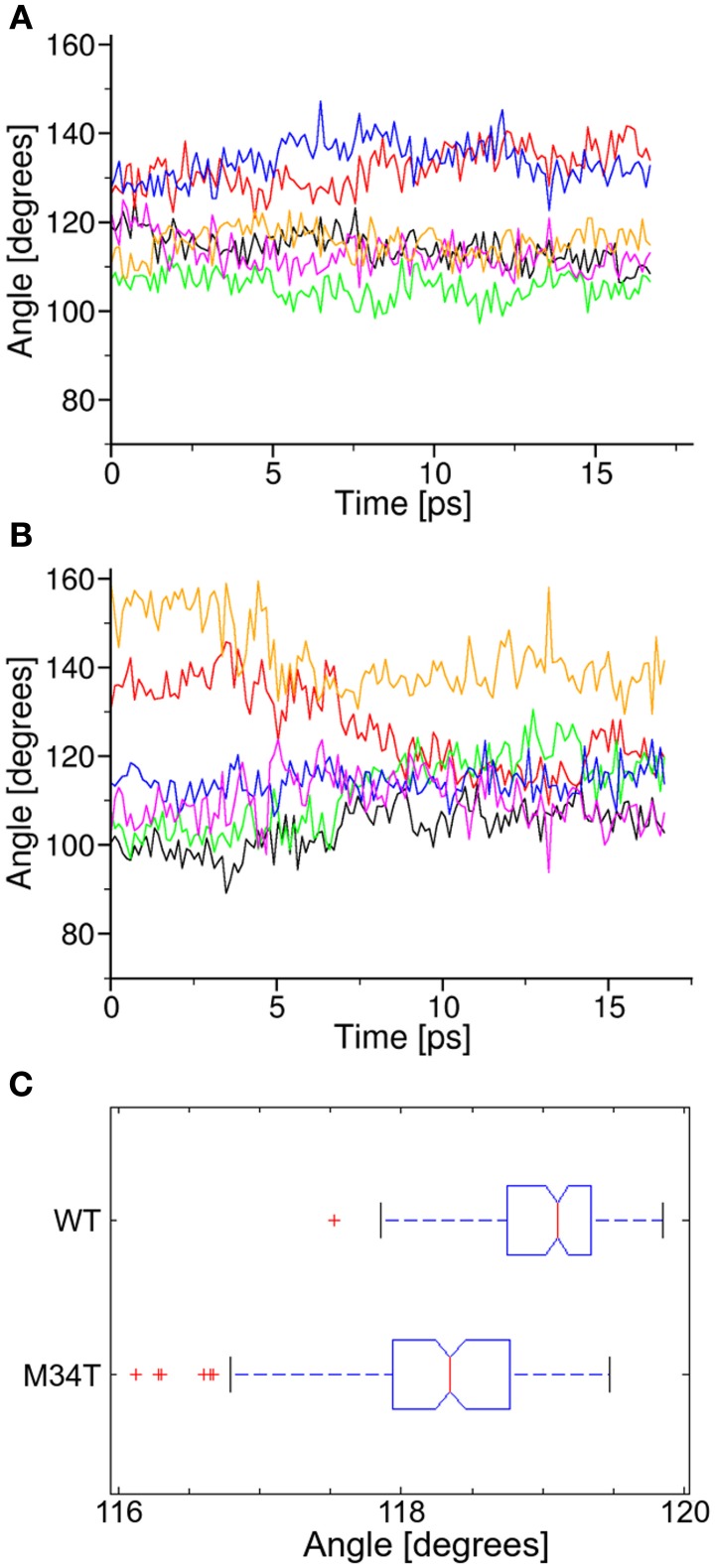
**Analysis of angular parameters**. Panels **(A,B)** show the time course of angular values during equilibrium dynamics for Cx26WT and Cx26M34T, respectively (see also Figure 3). **(C)** Box plots of the two data distribution, in which we interpreted each angle as a representation of the corresponding observable. The difference between the two distribution is significant (see text).

We further analyzed the dynamical behavior of the NTHs by tracking the angles of the hexagon built on the alpha carbon of the six T5 residues, which are located roughly half way along the NTHs. In Figure 5 we plot angular values during the last 18 ns of equilibrium dynamics for both Cx26WT (Figure 5A) and Cx26M34T (Figure 5B) vs. time. Note that angles in the Cx26M34T hemichannel display a higher degree of instability compared to Cx26WT and, correspondingly, the distribution of their values differ significantly (Figure 5C, *p* = 0.01, Mann–Whitney *U*-test).

### The M34T mutation reduces the interaction between the NTHs and the inner wall of the hemichannel

**Figure 6:**
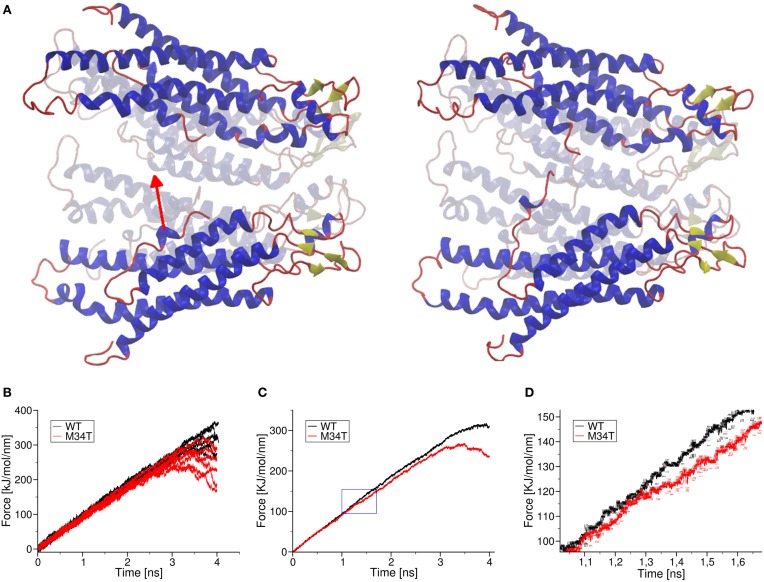
**Effect of a pull force applied to the NTHs**. Panel **(A)** shows schematically the effect of pulling one NTH: shown are the initial and final frames of a pulling simulation. In panel **(B)**, we plot raw pull force data for each trajectory corresponding to the six different helices of Cx26WT (black traces) and Cx26M34T (red traces). Panel **(C)** shows the mean traces obtained from the corresponding raw traces, after application of a further running average over 200 fs in order to reduce thermal noise. The blue box is magnified in panel **(D)** to show more clearly the point where the two mean traces separate. Error bars are standard deviation obtained from the running average. Visual inspection of the molecular dynamics trajectories revealed that, in the mutant, the detached helix interacts with a neighboring NTH, due to the more asymmetric shape of the pore mouth. This interaction obstacles the motion of the helix toward the center, until the pull force is large enough to break it. This effect was not observed in wild the type Cx26WT (Supplementary Movie S3).

It has been proposed that, for a gap junction channel to reside in the fully open state, the six NTHs must be attached to the inner wall of its cytoplasmic mouth via hydrophobic interactions between W3 and M34 (Maeda and Tsukihara, 2011; Fasciani et al., 2013). To test this hypothesis, we performed a series of steered molecular dynamics simulations by connecting the center of mass of one NTH (residues 1 to 12) to one end of a linear spring with zero resting length and elastic constant of 100 kJ mol^−1^nm^−2^ (Figure 6). Figure 6B shows six different pull force traces (one per NTH) for Cx26WT (black) and Cx26M34T (red). At the beginning of each run, the two spring ends coincided and the pull force was null. Setting the free end of the spring into motion with constant velocity of 1 nm ns^−1^ toward the center of the pore generated a centripetal force on the NTH. In the typical scenario, the pulled NTH did not follow immediately because the pull force was insufficient to overcome the interaction that kept the NTH attached to the inner wall of the hemichannel. Consequently the spring extended and the pull force increased linearly, until it reached a value sufficient to break the NTH–wall interaction. At this point the NTH started to move, the spring relaxed and the pull force dropped abruptly. In the Cx26M34T hemichannel the pulled NTH started to move after about 1 ns, whereas in the Cx26WT hemichannel the movement occurred at the end of the simulation period (after ~3 ns). To highlight the differences between the two data sets, we averaged the six different traces and computed a running average over these mean traces to reduce the effect of thermal noise (Figure 6C). Note the clear departure of the average traces around 105 (kJ/mol)/nm (Figure 6D). We interpret this value as the detaching force for the Cx26M34T mutant. The corresponding value for Cx26M34T was in excess of 250 (kJ/mol)/nm, almost three–fold larger.

## Discussion

At the cellular level, the deafness–associated Cx26M34T mutant is correctly synthesized and targeted to the plasma membrane in HeLa cells, but inefficiently forms intercellular channels that display an abnormal electrical behavior and retain only 11% of the unitary conductance of Cx26WT (Bicego et al., 2006). Moreover, Cx26M34T channels fail to support the intercellular diffusion of fluorescent tracers and the spreading of mechanically induced intercellular Ca^2+^ waves (Bicego et al., 2006). It has also been suggested that Cx26M34T/Cx32 heterotypic channels are not fully open at rest but are activated when positive transjunctional voltages are applied to the Cx26M34T side (Skerrett et al., 2004).

Our molecular dynamics simulations of Cx26WT and Cx26M34T hemichannels indicate that the quaternary structure of the mutant is altered at the level of the NTHs due to the disruption of the hydrophobic interaction between M34 (in the first transmembrane helix) and W3 (in the NTH) (Figure 3). The mutation destabilizes the NTH binding to the cytoplasmic mouth of the channel altering its shape, which is significantly more asymmetric in the mutant hemichannel model compared to the wild type model (Figures 4, 5).

The NTHs are thought to participate in voltage gating, which is rather complex in channels formed by human Cx26 that exhibit a bipolar behavior (Gonzalez et al., 2006). Single channel recordings indicate that the opening of Cx26 hemichannels upon depolarization at negative potentials involves a transition from the fully closed state to a main open state. As depolarization progresses, hemichannels remain stable in this high conductance state until polarization reaches larger positive potentials, whereupon they inactivate by closing partially to a subconductance or residual open state (Gonzalez et al., 2006).

To explain these observations, it has been proposed that connexin hemichannels possess two gating mechanisms. One mechanism has the same polarity for all the hemichannels independently of their connexin isoform composition and depends critically on the extracellular Ca^2+^ concentration (Ebihara and Steiner, 1993; Pfahnl and Dahl, 1999; Muller et al., 2002). A study performed on Cx32 hemichannels concluded that (i) Ca^2+^ can block both voltage gated opening to the higher conductance open state and ion conduction through the partially open hemichannels and (ii) the effect depends on Ca^2+^ binding with millimolar affinity within the extracellular vestibule of the pore (Gomez-Hernandez et al., 2003). In our prior work using molecular dynamics simulations, we provided an interpretative model showing that Ca^2+^ ions linger within the negatively charged extracellular mouth at a membrane potential of −80 mV. Upon depolarization to 0 mV the interactions weaken and the position of the Ca^2+^ ions shifts significantly toward the extracellular space (Zonta et al., 2012). This scheme is supported by the presence of negatively charged amino acids facing the pore in the extracellular mouth, in particular D46 and E47, that are highly conserved across connexin isoforms. E47 is also believed to undergo post-translational gamma carboxylation (Locke et al., 2009), which increases it affinity for Ca^2+^ ions. This crucial point is analyzed in Zonta et al. (2014).

The second gating mechanism shows different polarity among different connexin isoforms, and depends on the total charge of the NTHs (Verselis et al., 1994). It has been proposed that the NTHs move in response to changes in electrical potential and close the channel (Maeda and Tsukihara, 2011; Fasciani et al., 2013). Our simulations indicate that the NTHs in the Cx26M34T mutant are less bound to the channel wall (Figure 6) and we speculate that, for this reason, the gating mechanism is compromised, as proposed in Skerrett et al. (2004). The slight but significant modifications highlighted by our relatively brief (40 ns) equilibrium molecular dynamics simulations, reflect in a sizeable (90%) reduction of the unitary conductance (Table 1), in quantitative accord with the experimental results (Bicego et al., 2006). Our simulation work also agree with the proposal that the M34T mutant channels resides most of the time in a low conductance state (13 pS for Cx26M34T, 15–20 pS for Cx32M34T). However, the displacement of the NTHs in our equilibrium dynamics, is not marked enough to be compatible with the formation of the pore plug as described in Oshima et al. (2007) for the closely related Cx26M34A mutant. Further simulation work is required to test whether such a plug can be formed by Cx26M34T mutant connexins.

### Conflict of interest statement

The authors declare that the research was conducted in the absence of any commercial or financial relationships that could be construed as a potential conflict of interest.

## Supplementary materials

The Supplementary Material for this article can be found on line at: http://www.frontiersin.org/journal/10.3389/fphys.2014.00085/abstract

Supplementary Movie S1 **Steered molecular dynamics of K^+^ permeation**. In this simulation, we force a K^+^ ion to permeate through the pore of Cx26WT and Cx26M34T hemichannels. The total PMFs for the two cases (black trace for wild type and red trace for the mutant) is shown in register with the permeating ion position (represented with its Van der Waals radius, in pink). The NTHs are highlighted in red, and two key residues are represented in ball and stick; T34 is color in green and K41 (corresponding to the maximum of the total PMF) in orange.

Supplementary Movie S2 **Steered molecular dynamics of K^+^ permeation seen from the cytoplasmic point of view**. The movie compares wild type (on the left) and mutant hemichannel (on the right). See also the caption of Figure 2.

Supplementary Movie S3 **Effect of pulling one NTH toward the center of the pore**. The pulled helix is colored in red, while the other NTHs in blue. The initial position is shown as a reference in white. The pulling of a helix in the wild type (on the left) and in the mutant (on the right) reveals different qualitative behaviors, in addition to the quantitative differences described in Figure 6. While in the wild type hemichannel the pulled helix moves abruptly at the end of the simulation, for the mutant this motion occurs earlier, but is then hampered by the interaction of the pulled helix with a neighboring NTH which are more mobile and do not keep the symmetric configuration of the wild type.
